# Does green credit promote real economic development? Dual empirical evidence of scale and efficiency

**DOI:** 10.1371/journal.pone.0326961

**Published:** 2025-08-05

**Authors:** Song Jiang, Xiaoqin Yuan

**Affiliations:** 1 School of Economics and Finance, Chongqing University of Technology, Chongqing, China; 2 Post-Doctoral Scientific Research Workstation of Bank of Chongqing, Chongqing, China; University of Almeria: Universidad de Almeria, SPAIN

## Abstract

Green credit has emerged as a new engine for economic transformation and a core driving force behind high-quality development. By using panel data from 30 provinces in China, this paper comprehensively examines the influence of green credit on the real economy. The research findings suggest that green credit has a significantly positive effect on the scale of the real economy but a significantly negative effect on the efficiency of the real economy. In addition, we construct instrumental variables for green credit to address endogeneity problems, The results are tested by rigorous robustness checks. The mechanism analysis reveals that green credit mainly exerts its influence by promoting the optimization of the industrial structure, reducing energy consumption and reducing green innovation. The reduction of green innovation patents due to green credit undoubtedly reflects significant obstacles in the current process of promoting economic transformation through green credit in China. This study provides a theoretical framework for understanding the macro-level implications of green credit. Moreover, it offers valuable insights for formulating policies targeting the financial services sector of the real economy.

## 1. Introduction

In the contemporary era characterized by rapid technological advancements, the real economy remains pivotal in the economic landscape [1]. The real economy not only directly supplies products and services to society, but also serves as the primary source of employment for the labor force [2]. This promotes resident jobs and contributes to social stability and harmony. Moreover, by providing intermediate products, the economy drives the development of related industrial chains, facilitates industrial upgrading, and supports a country’s economic transformation and high-quality development.

However, the traditional economic development model is unsustainable and needs to be transformed urgently. Many countries, led by the United States and Japan, have adopted the “pollute first, treat later” approach to achieve rapid economic growth. This has resulted in resource shortages, an intensification of the greenhouse effect, and severe pollution problems [3]. The carbon emissions from the extensive consumption of fossil fuels have exacerbated global climate change, significantly impacting environmental quality [4]. Studies have indicated that the adverse effects of climate change, such as rising sea levels, declining agricultural yields, deteriorating air quality, freshwater shortages, extreme weather events, and ecological degradation, pose threats to human physical and mental health, social stability, and sustainable development [5]. These impacts are felt globally, and in emerging and industrialized economies like China, which have high demands for energy and resources, these challenges endanger the sustained and robust growth of the real economy. Consequently, there is an urgent need to transform and upgrade the traditional real economy model.

China is facing tremendous pressure for economic transformation. As a typical developing country, China has achieved remarkable economic progress over the past four decades of reform and opening, with a significant increase in its comprehensive national strength. However, objectively, the rapid growth in the past few decades was based on a crude development model that relied on large-scale consumption of material resources and pursued quantitative and spatial expansion. This growth path overemphasized economic growth’s speed while neglecting its quality, leading to numerous resource, environmental, ecological, and social issues, such as tightening resource constraints, severe environmental damage, and economic inefficiency. With profound changes in the international market environment and domestic factor endowment conditions in recent years, the previous high input, high consumption, and high pollution crude development model is no longer sustainable. There is an urgent need to transform the economic development model from a crude one focused on factor inputs to a high-quality model that integrates environmental protection and economic efficiency.

Finance, as the lifeblood of the real economy, is a crucial factor in promoting innovative development and efficiency. Financial reform and innovation are crucial in transitioning from a high- to a low-carbon economic model. To achieve the green goal, it is necessary to strengthen end-to-end governance and adjust the incentive mechanism of resource allocation through financial and other means. In this context, green finance has emerged and is continuously evolving. Against the backdrop of reaching the carbon emissions peak goal, green management has become a new driving force for economic transformation and a key enabler of high-quality development. Green finance has been widely recognized as an important financial instrument to support sustainable economic development. It has been integrated into financial institutions at all levels, from concepts and strategies to product innovation, and has become a development trend for modern financial institutions. As more economies set higher standards for green finance in line with relevant policies and regulations, it will become increasingly difficult for high-carbon emitting and heavily polluting industries to obtain sufficient support from green finance.

Green finance encompasses various forms, including green credit, securities, insurance, investment, and carbon finance. Given the structure of China’s bank-dominated financial system, green credit plays a dominant and important role. The core of green credit lies in promoting environmental improvement, combating climate change, and facilitating the effective use of resources. It contributes to environmental protection and guides enterprises from highly polluting and energy-intensive industries towards a more sustainable direction. Examining the path of green credit’s impact on economic development while mitigating environmental deterioration is crucial for both green credit and high-quality economic development.

The release of the “Opinions on the Implementation of Environmental Protection Policies and Regulations for the Prevention of Credit Risks” in 2007 marked the official implementation of green credit, an environmentally oriented financing policy, in China. According to data released by the People’s Bank of China, by the end of 2022, the balance of local and foreign currency green credit in China reached RMB 22.03 trillion, a year-on-year increase of 38.5%, 5.5 percentage points higher than at the end of the previous year, and 28.1 percentage points higher than the loan growth rate, with an annual increase of RMB 6.01 trillion.

However, alongside hopes lie obstacles. Compared with international experience, China’s overall green credit is still in its infancy. Green financial policies incur implementation costs, and banks’ green governance costs may be passed on to the real economy through various credit channels. This may distort the allocation of social resources and deviate from the original intention of green credit innovation. Banks implementing green governance are generally more concerned about the environmental performance of borrowers, and their loan pricing is more sensitive to environmental risks, resulting in higher credit rates. There is a prevalent incentive for firms to engage in “greenwashing” at the demand level. Firms carry out green innovation merely to obtain government subsidies and reduce social regulatory pressures rather than to reduce negative environmental impacts and improve environmental performance. Providing green financial services to these “greenwashing” enterprises will, to some extent, increase the credit risk of commercial banks, making it highly uncertain whether green credit can achieve the desired environmental effects.

Moreover, weak institutions and regulatory frameworks can hinder the effective implementation of green credit policies. In many developing countries, including China, inconsistent enforcement of environmental regulations and underdeveloped governance structures reduce the credibility and effectiveness of green finance initiatives. From the perspective of institutional economics, unclear property rights and an inadequate legal framework limit the enforceability of green loan contracts and accountability of borrowers. In summary, green credit does not only bring positive impacts on real economic development; its ultimate outcomes still warrant further research. Currently, there is a lack of in-depth studies in this area.

Therefore, whether green credit can promote the growth of the real economy and improve the efficiency of the real economy in China depends on whether green credit funds can be efficiently allocated among enterprises and between different projects within enterprises. So, this paper tries to answer the following questions: how does green credit affect the scale and efficiency of real economic development? What are the channels through which these effects occur? And to what extent do the impacts vary across provinces and cities? By addressing these questions, this study aims to clarify the mechanisms through which green credit influences China’s economic development, as well as to identify the obstacles that may hinder its effectiveness in promoting sustainable growth.

The significance of this study lies in constructing a theoretical, analytical framework for green credit and actual economic development. This framework can deeply uncover the mechanism by which green credit influences real economic development, providing a theoretical basis for empirical testing and establishing an academic, theoretical, and discourse system to support real economic development. Simultaneously, through multi-dimensional econometric tests, this study assesses the deviation between actual practice and theoretical expectations, identifies the current constraints of green credit on real economy development, and delves into the reasons for such deviations. This provides decision-making support for promoting the adjustment of green credit policies to support real economic development.

The potential marginal contributions of this study can be summarized as follows: (1) Theoretically, it deepens the understanding of the relationship between green credit and the real economy, especially within China’s bank-dominated financial system. (2) Methodologically, it calculates the efficiency of the real economy in 30 Chinese provinces from 2007 to 2022 using the Super-SBM model. (3) In terms of empirical analysis, it explores the relationship between green credit and the real economy. It examines how green credit affects real economic development via upgrading industrial structures, promoting green innovation, and optimizing energy consumption structures. It also distinguishes between real economy development’s scale and efficiency effects, offering a new direction for subsequent research. (4) In analyzing the heterogeneity of green credit’s impact on the real economy, it explores this impact from the dimensions of the region, the development level of the banking system, and environmental regulations, providing more comprehensive and systematic theoretical support for decision-making. The core purpose of this study is to empirically reveal the impact of green credit on real economy development, clarify its mechanism and support path, integrate research data to conclude, and provide a theoretical basis and reference for government decision-making.

The remainder of the study is structured as follows. Section 2 reviews relevant literature and formulates research hypotheses. Section 3 details the technical process, research methods, and data sources. Section 4 evaluates the effect of green credit using a benchmark model. Section 5 comprehensively discusses green credit’s institutional pathways and heterogeneity for real economic development. The final section summarizes the research findings, analyzes the limitations of this study, and suggests directions for future research.

## 2. Literature review and research hypotheses

### 2.1 Literature review

#### 2.1.1 Green credit-related studies.

As environmental issues have increasingly become a global consensus in recent years, the demand for finance for environmental governance has been growing. As an important part of China’s green financial system and the most important green financial product, green credit has triggered widespread attention. Currently, academic research on green credit mainly focuses on the environmental and economic effects of green credit. Regarding the environmental effect of green credit, the research mainly focuses on micro-enterprises and barriers to realization. Firstly, regarding the impact on enterprises, the green credit policy requires the banking sector to reduce its credit exposure to the “two high and one surplus” industries and reduce energy consumption by controlling external financing to realize environmental benefits. The policy significantly improved the ease of access to finance for green-listed firms but exacerbated the financing penalties and investment disincentives for heavily polluting firms. An increase in credit funding implies an increase in external financing sources for firms, which positively affects the technological innovation of listed firms [6]. The appropriate environmental regulation can stimulate firms to innovate, reduce environmental costs through technological innovation, improve firms’ environmental performance, and increase firms’ productivity and competitiveness [7].

This positive incentive helps enterprises to improve their green innovation ability, pay more attention to energy saving and emission reduction in the production and operation process, and ultimately realize the green transformation of economic development. The technological progress of carbon emissions has an inhibitory effect on industry and region [8]. In addition, given the multi-dimensional impacts of green credit, scholars have thoroughly examined the real-world obstacles and practical challenges encountered in realizing its environmental benefits. Green credit will be challenged by the lack of environmental information in the process of its implementation, the inadequacy of the supporting policies and regulations, the lack of clarity of the industry implementation standards, local protectionism, and other challenges [9,10]. Unclear, local protectionism, and other challenges. In addition, other scholars believe that the realization of the environmental effects of green credit policy is also affected by the nature of enterprises with different ownership systems. The green credit policy requires commercial banks to take the environmental compliance of enterprises as a mandatory condition for approving green loans and to refuse or strictly limit the issuance of loans to polluting enterprises. Thus, study reveals that the environmental effects of commercial banks’ green credits differ significantly across ownership firms [11]. Specifically, credit discrimination is prevalent in the supply of bank credit due to multiple factors, such as government intervention [12], resulting in most credit resources flowing to state-owned enterprises, while private enterprises are often difficult to access [13].

In terms of the economic ramifications of green credit, these have been shown to primarily impact two levels: namely, the macroeconomic development and industrial structure. Regarding macroeconomic benefits, scholars have argued that implementing green supply policies significantly promotes economic development and positively affects industries and intra-industries. Asymmetric input of financial sector-led capital resources between sunrise and sunset industries can enhance resource allocation efficiency [14]. Furthermore, commercial banks and other financial institutions incorporate resource conservation and pollutant emission control into the green credit assessment process, creating a “signaling function” that provides an effective means for macroeconomic growth [15]. In addition to focusing on the macro effect, scholars also start from the level of industrial structure, focusing on the structural effect formed by green credit. Green finance, as a form of financial innovation, effectively engages with the optimization and upgrading of industrial structures, and those green financial instruments, such as green credit, facilitate the effective allocation of funds to the green and environmental protection industry, thereby promoting the decarbonization of industrial structures [16]. In addition, green finance fosters the emergence of alternative and novel energy sources by developing innovative financial instruments while concurrently imposing stringent regulations on promoting high-pollution and high-emission projects [17]. This regulatory framework optimizes the industrial structure and promotes the transition of the energy consumption structure towards clean energy sources.

#### 2.1.2 Relevant studies on the real economy.

In economics, scholars have reached a consensus regarding classifying factors that influence the real economy. These factors are typically divided into macro, meso, and micro. Macro-level factors are understood to encompass economic growth, policies [18], and shocks [19–21]. Frequent economic policy adjustments engender economic policy uncertainty and exert a contractionary effect on real investment, while credit plays a pivotal role as the primary financial conduit for transmitting policy uncertainty shocks to the real economy [22].

Focusing on meso-level factors, study [23] examined the African real economy, concluding that equity portfolio investments do not stimulate growth in the real sector of the African economy. However, they determined that debt flows impede real, manufacturing, and industrial growth.

Conversely, financial development was found to reinforce the positive correlation between capital flows and economic growth. Researchers conducted a study [24] utilizing data from the real economy of Turkey, which revealed that the volume of lending in the banking sector, particularly Islamic banking, exerts an influence on the sector under consideration. Furthermore, the causal relationship exhibited by this phenomenon is subject to alteration over time. Concerning micro-factors, the predominant elements pertain to the economic activities of banks [25,26] and firms [27].

The role of financial development in enhancing both the scale and efficiency of the real economy has also been widely studied. Study [28] demonstrated that well-developed financial markets improve capital allocation, support entrepreneurship, and promote long-term growth by reducing transaction costs and facilitating risk-sharing. Study [29] further showed that access to finance is particularly important for small and medium enterprises, which are critical contributors to employment and innovation in the real economy.

More recently, attention has shifted to the role of green finance, especially green credit, in promoting sustainable economic development. Green credit can align financial flows with environmental goals, supporting the transition toward low-carbon industries without sacrificing economic growth. Empirical studies have found that green credit policies can encourage firms to adopt cleaner technologies, reduce emissions, and improve operational efficiency. Evidence from China showing that green credit policies significantly improve corporate environmental performance, although challenges remain in terms of implementation and incentive design.

In summary, while traditional economic theories emphasize capital, labor, and technology as the primary drivers of real economic development, recent research highlights the growing importance of institutional quality, financial intermediation, and environmental sustainability. The emergence of green credit reflects a broader shift toward integrating ecological considerations into economic policy and financial decision-making.

#### 2.1.3 Literature review.

The extant literature on the subject is inconclusive, with a paucity of studies that have analyzed the impact of green credit on real economic development. Nevertheless, many scholars have conducted in-depth research on how green credit fosters economic growth, primarily at the level of micro-enterprises; green credit functions as an external factor in addressing the issue of environmental pollution. It catalyzes enterprises to allocate capital towards developing environmental technology and the innovation of new green products, thereby transforming their investment and production behavior and propelling them towards a sustainable development trajectory. Enterprises can enhance their competitive advantage, expand profit growth, optimize investment strategies, and enhance technological innovation, environmental awareness, and other practices. This, in turn, can enhance production efficiency and competitiveness, thereby promoting the development of the real economy. From a macroeconomic perspective, numerous scholars posit that implementing a green credit policy is conducive to the development of the entire nation. According to Schumpeter’s economic development theory, credit capital will spontaneously flow to areas consistent with the direction of social development, promote industrial upgrading, lead technological progress, and drive economic development.

The extant research literature about green credit and economic growth provides valuable references and research ideas for this paper, which explores the influence mechanism and path support of green credit on real economic development. However, these studies are also afflicted by certain deficiencies. Primarily, China belongs to the bank-led financial system, and green credit, as the most important component of the green financial system, needs to be further explored in depth. Moreover, in studying the economic effect of green credit, the empirical study of the macroeconomic effect on the development of the real economy and other macroeconomic effects is still insufficient. In the context of high-quality development of the economy, the demand for research in this area is particularly urgent. Secondly, extant research on the mechanism of green credit is inadequate, particularly research on the influence mechanism and support path of real economic development. This project aims to empirically study the mechanism of green credit and real economic development, reveal the influence mechanism and support path of green credit on real economic development, integrate research data to drive conclusions, and provide theoretical support and decision-making basis for the government.

### 2.2 Theoretical analysis and research hypothesis

#### 2.2.1 Direct impact of green credit on real economic development.

The direct impact of green credit on the development of the real economy is mainly reflected at the macro and micro levels—first, the macro level. By implementing differentiated monetary policy, such as credit tilt, interest rate fluctuations, and other means, green credit promotes capital pooling and forming green investment, providing capital elements for economic growth [8] Green credit policy requires commercial banks to fully consider the environmental risks of loan projects in the loan decision-making process [30], strengthen financial support for enterprises that meet internationally recognized green financial standards while cutting financial support for highly polluting entities, and reach a green allocation of credit resources, which will help to reduce the sources of pollution, and thus improve environmental quality. The lower financing cost of green projects prompts the flow of funds to efficient, energy-saving, low-pollution green projects, which will promote the optimization of economic structure and the improvement of the quality of economic growth; green credit is conducive to guiding capital inflow into the green industry, promoting the development of green industry and related emerging industries, and forming a new point of economic growth.

The second is the micro level. Green credit, with its specialized information collection, analysis, and evaluation capabilities, screens and selects potential investment projects and presents green projects with investment value and environmental benefits to the public and investors, thus driving the optimal allocation of social resources and real economic growth. In addition, the government publishes information such as the list of polluting enterprises and environmental credit records, which can mitigate adverse selection and moral hazard and reduce the cost for investors to obtain information on their own. In addition, enterprises often need to invest much capital in green technological innovation, facing more significant risks. In the initial stage of green technological innovation, traditional finance is complex to play a role and cannot bring direct economic benefits for enterprises. Green credit can provide sufficient financial support for the research and development, transformation, and application of green technology, thus reducing the innovation risk of enterprises. Finally, to ensure the safe recovery of funds and their interests, the financial institutions implementing green credit carry out strict qualification audits of the projects or enterprises applying for loans through their environmental risk identification and assessment mechanisms and carry out timely follow-up on the use of funds and energy-saving and emission reduction effects of the enterprises after the loans are granted. For enterprises with sound emission reduction effects, financial institutions can appropriately increase the loan amount; for enterprises with poor rectification, they will stop lending to improve the efficiency of capital allocation and promote economic growth.

However, the neoclassical school of economics argues that environmental policies may harm economic growth by raising private production costs and weakening the competitiveness of firms, offsetting the positive effects of environmental protection on society. As a specific form of environmental regulation, the possible negative impact of green credit on economic performance cannot be ignored. According to the cost regulation theory, environmental regulation is tantamount to imposing additional constraints on a firm’s decision-making process [31,32], which makes it more difficult and costly for firms to produce, sell, and manage their activities. Implementing green credits increases the financing constraints of heavily polluting firms that require significant capital investments for equipment purchases, R&D activities, and process upgrades, resulting in higher loan costs and reduced financing for these firms. Enterprises thus bear higher risks and sunk costs, which may force them to reduce capital investment in R&D, production, and investment activities, which in turn leads to the loss of market share and competitive advantage, impairing their profitability and productivity, and is not conducive to the improvement of total factor productivity [33,34]. Study [35] stated that while environmental regulation can improve environmental conditions, it may simultaneously bring about unnecessary production costs and limit firms’ productivity. The findings [36] suggest that the costs of environmental regulation may outweigh the benefits it brings, negatively affecting firms’ productivity. In addition, study [37] found that financing abatement policies represented by green credits significantly and punitively affect total factor productivity, profitability, and sales growth of highly polluting firms. Based on the above analysis, we propose the following hypothesis.

H1: Green credit positively affects the size of the real economy.

H2: The impact of green credit on the efficiency of the real economy is uncertain and may increase or decrease.

#### 2.2.2 Indirect impact of green credit on real economic development.

The literature review shows that although previous literature has not directly explored the direct impact of green credit on real economic development, studies on the impact effects of green credit provide a basis for constructing a theoretical framework. Based on these findings, this study proposes the hypothesis that green credit promotes the development of the real economy through three paths: industrial structure upgrading, green innovation, and energy consumption reduction.

**2.2.2.1 Green credit and industrial structural upgrading:** Green credit takes the environmental performance of enterprises as the evaluation standard and precisely regulates the scale and direction of credit funds. This mechanism aggregates idle funds in society through savings, provides low-cost financial support for green projects, and thus promotes enterprises to progress in green production, energy-saving transformation, and upgrading. In addition, green credit restricts polluting enterprises’ financing, raises operating costs, and increases the pressure on their survival, forcing them to either make technological innovations or withdraw from the market. Both scenarios will have a far-reaching impact on the local industrial structure and promote its transformation towards greening and upgrading. This process not only improves the quality of financial services to the real economy and helps to correct the mismatch of social resources but also transforms the traditional model of crude economic development, promotes the efficient use of resources and environmental protection, which in turn leads to higher output, improved product quality and added value, and enhanced economic efficiency. At the same time, optimizing industrial structure also helps improve the efficiency of the real economy. With green credit guiding the flow and distribution of funds and resources, combined with the government’s leading role, the environmental awareness of enterprises has been strengthened. Those traditional industries that do not meet the development needs of the times, with high input, high pollution, and low efficiency, will be gradually replaced by high-efficiency industries, thus accelerating the construction of a modernized industrial system and realizing the optimization and upgrading of the industrial structure and the effective enhancement of productivity. Thus, we propose our third hypothesis.

H3: Green credit promotes the real economy by upgrading the industrial structure.

**2.2.2.2 Green credit and green innovation:** Based on the endogenous growth theory of R&D, this study considers technological advances in environmental protection as a key source of sustainable economic growth. Investing in green innovation differs from other forms of investment. It involves high uncertainty and significant upfront capital requirements that are difficult to meet with internal financing alone. As an important tool for the banking sector in promoting environmental protection and economic growth, green credit is crucial in stimulating green innovation among companies. Green credit can effectively alleviate the financing constraints of green innovation by providing investments and loans to support the development of clean technologies and reduce the cost of capital for such initiatives. Providing low-interest loans to enterprises engaged in environmentally friendly projects and increasing credit support for green projects expands the financing channels of green and clean enterprises. It enhances the internal drive for technological innovation, which in turn promotes the development of green technologies. Moderate environmental regulation can stimulate enterprises to realize technological advancements in production and environmental protection upgrading, improving resource use efficiency and enhancing the development of green technologies. By restricting or denying financing for non-green projects, green credit prompts firms to increase their environmental expenditures to obtain loans. Rising environmental costs increase the pressure on firms, which may incentivize firms to seek technological innovations to improve productivity to compensate for the increased environmental costs, thus indirectly forcing polluting firms to make green technological innovations. In addition, green credits increase the level of environmental disclosure by firms, encouraging them to fulfill their social responsibilities by increasing market transparency and promoting more incredible innovation and research in green patents. This not only helps build a positive brand image but also attracts the attention of investors and consumers [38,39]. Therefore, green credit is an important catalyst to promote green technological advancement and helps to promote sustainable economic development. However, alongside hopes lie obstacles. One the one hand, green projects often require high upfront investments and offer long payback periods, which may not yield immediate financial returns. This can make both firms and lenders cautious about engaging in green credit programs, especially in the absence of sufficient policy support or risk mitigation mechanisms. On the other hand, weak institutions and regulatory frameworks can hinder the effective implementation of green credit policies. In many developing countries, including China, inconsistent enforcement of environmental regulations and underdeveloped governance structures reduce the credibility and effectiveness of green finance initiatives. From the perspective of institutional economics, unclear property rights and an inadequate legal framework limit the enforceability of green loan contracts and accountability of borrowers. Therefore, we propose our fourth hypothesis.

H4: Green credit stimulates or hinders the real economy by incentivizing or reducing green innovation.

**2.2.2.3 Green credits and energy consumption:** A substantial body of research has demonstrated the considerable potential of green credit in facilitating the transition of highly polluting industries to clean energy sources. By offering preferential loans and credit support, green credit incentivizes firms to adopt clean energy and energy-efficient technologies, reducing their reliance on high-carbon energy sources such as coal. Concurrently, the direction of credit policy prompts financial institutions to impose credit restrictions on high-pollution and high-energy-consumption projects, compelling enterprises to enhance their energy structure and efficiency. For instance, financial institutions can offer reduced preferential lending rates for enterprises investing in renewable energy projects, such as solar and wind power, or provide additional credit lines for enterprises with significantly improved energy efficiency. Implementing these preferential loans and credit support measures has led enterprises to increasingly opt for renewable energy sources such as solar and wind power, thereby reducing their reliance on fossil fuels. This transition has not only contributed to a reduction in greenhouse gas emissions but also enhanced energy efficiency and fostered the growth of the green economy. Moreover, green credit can catalyze consumers and enterprises to modify their energy consumption habits, popularize the use of energy-saving products, and further promote the sustainable development of the real economy. Implementing these measures has assisted enterprises in reducing operating costs while driving the transformation of the social energy consumption structure toward a more sustainable and efficient model. Concurrently, implementing green credit policies can stimulate market demand for green technologies and services, thereby promoting the prosperity of associated industries and establishing a virtuous cycle. Hence, we propose our last hypothesis.

H5: Green credit promotes the real economy by reducing energy consumption.

**Fig 1:**
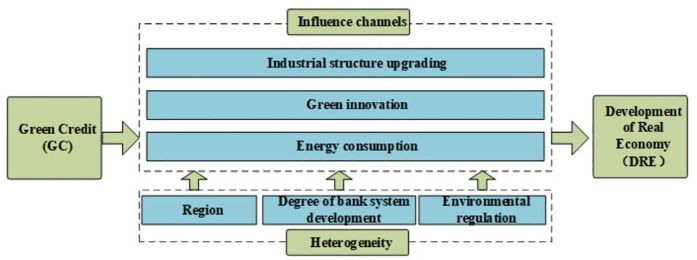
Theoretical analysis framework.

The above theoretical analysis route is shown in Fig 1.

## 3. Research design

### 3.1 Model specification

#### 3.1.1 Benchmark regression model.

To test the effect of GC on SRE and REE as well as heterogeneity, two-way fixed-effects models (1) and (2) were developed concerning the model settings:


$$SRE_{it}=\alpha_{0}+\alpha_{1}GC_{it}+\alpha_{2}Controls_{it}+\mu_{i}+\gamma_{t}+\varepsilon_{it}$$ (1)

$$REE_{it}=\beta_{0}+\beta_{1}GC_{it}+\beta_{2}Controls_{it}+\mu_{i}+\gamma_{t}+\varepsilon_{it}$$ (2)

Where i stands for up to provinces, t stands for up to years, $REGDP_{it}$ is t year i region real economy size, $REE_{it}$ is t year i region real economy efficiency, $GC_{it}$ is t year i region green credit development level, $Controls_{it}$ is the set of control variables, μ is the region fixed effect, γ is the year fixed effect, and ε is the random error term. This paper focuses on the sign of $\alpha_{1}$ and $\beta_{1}$ and their significance, i.e., the effect of GC on SRE and REE.

#### 3.1.2 Mediated effects model.

To further explore the channels of how green credit affect the development of the real economy, we test the potential mechanisms. First, we check the link between green credit and the channel variable M. Second, we check the relationship between the mechanism variable M and actual economic development. The specific regression model is as follows:


$$REGDP_{it}=\alpha_{0}+\alpha_{1}GC_{it}+\alpha_{2}Controls_{it}+\mu_{i}+\gamma_{t}+\varepsilon_{it}$$ (3)

$$REE_{it}=\beta_{0}+\beta_{1}GC_{it}+\beta_{2}Controls_{it}+\beta_{3}M_{it}+\mu_{i}+\gamma_{t}+\varepsilon_{it}$$ (4)

$$M_{it}=\delta_{0}+\delta_{1}{GC}_{it}+\delta_{1}{Controls}_{it}+\mu_{i}+\gamma_{i}+\varepsilon_{it}$$ (5)

$$REGDP_{it}=\theta_{0}+\theta_{1}M_{it}+\theta_{1}Controls_{it}+\mu_{i}+\gamma_{t}+\varepsilon_{it}$$ (6)

$$REE_{it}=\eta_{0}+\eta_{1}M_{it}+\eta_{2}Controls_{it}+\mu_{i}+\gamma_{t}+\varepsilon_{it}$$ (7)

M refers to the mechanism variables that GC may affect in the real economy. Based on the above theoretical analysis, this study focuses on upgrading industrial structures, promoting green innovation, and promoting energy consumption.

#### 3.1.3 Super-SBM model.

The traditional CCR and BCC models have limitations in measuring the full range of slack variables. Given this, the SBM model, which effectively circumvents the effects of different radial and angular choices on efficiency values. To further accurately reflect the differences in real economic efficiency and its evolution trend among different provinces and regions, we will construct a Super-SBM model based on the SBM model to calculate the real economic efficiency. The specific Super-SBM model is as follows:


$$\begin{array}{c} \min\rho =\frac{1+\frac{1}{m}\sum {\mathrm{\backslash nolimits}}_{i=1}^{m}\frac{s_{i}^{-}}{x_{ik}}}{1-\frac{1}{s}\sum {\mathrm{\backslash nolimits}}_{r=1}^{s}\frac{s_{i}^{+}}{y_{rk}}}\\ \sum_{j=1,j\neq k}^{n}\lambda_{j}x_{ij}-s_{i}^{-}\leq x_{ik},i=1,2,\cdots ,m,\\ \sum_{i=1}^{n}\lambda_{j}y_{ri}+s_{r}^{+}\geq y_{k},r=1,2,\cdots ,s,\\ \lambda_{j}\geq 0,j=1,2,\cdots ,n(j\neq k),s_{i}^{-}\geq 0,s_{r}^{+}\geq 0, \end{array}$$

Where: ρ is the efficiency value, x, y is the input and output variables respectively; $s^{-},\;s^{+}$ is the input and output slack variables respectively; $\lambda_{j}$ is the vector of weights; m,s,n is the number of input and output indicators and the decision unit respectively.

### 3.2 Variables definition and data source

#### 3.2.1 Dependent variable.

This study defines the real economy as the sector of the economic system excluding the real estate and financial industries. The real economy is then analyzed quantitatively in terms of scale and efficiency. The utilization of the GDP per capita of the real economy facilitates the quantification of the real economy’s size. The real economy’s GDP per capita calculation is derived by deducting the value added of the financial and real estate sectors from the overall GDP, employing 2003 as the base year, adjusting the GDP deflator, and standardizing with year-end population data. Currently, there is a paucity of studies that measure the efficiency of China’s real economy from the perspective of total factor productivity. To accurately assess the development of China’s real economic efficiency and consider the availability of provincial data, this study adopts the efficiency value measured by the Data Envelopment Analysis (DEA) model as a representative indicator of regional real economic efficiency. The Super-SBM model is utilized to calculate the real economic efficiency of 30 provinces in China from 2007 to 2022. The model utilizes input indicators encompassing the real economy labor force and capital stock, with the output indicator designated as the real domestic GDP. The specific measurement formula is shown in Table 1.

**Table 1:** Description of input-output indicator.

Input/Output	Input/Output	Indicator Description
Input	Labor input	Sum of the number of unit employees, private employees, and self-employed employees in each city/10,000 people
	Capital input	taking 2003 as the base period and using the perpetual inventory method to calculate the capital input, the depreciation rate was set at 1096%/billion CNY. rate was set at 10.96%/billion CNY
Output	Real GDP	Real GDP – (Finance GDP + Real estate GDP) (based on 2003) (billion CNY)

**Fig 2:**
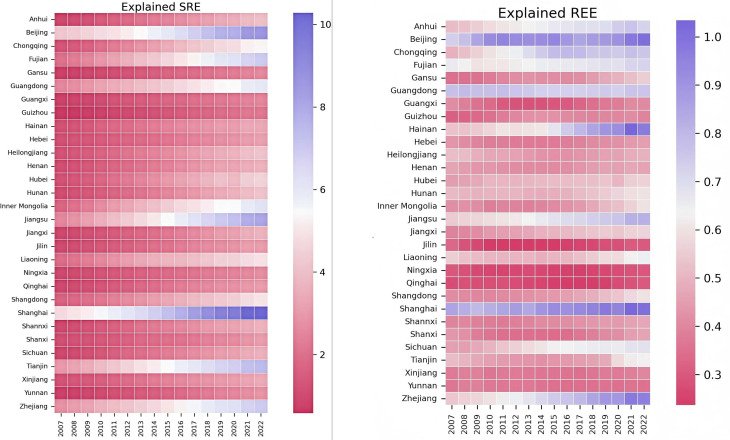
Distribution of DREs. Note: The data in Fig 2 are averages of SRE and REE based on province and year. Red indicates poorer real economic development, while purple indicates better development.

As demonstrated in Fig 2, which presents the evolution of DRE by the province in China from 2007 to 2022, the nation’s real economy has exhibited sustained growth. Among the provinces, Beijing, Shanghai, Zhejiang, and Jiangsu have exhibited notable actual economic development, while provinces such as Ningxia, Qinghai, Jilin, Guangdong, and Guangxi have demonstrated comparatively lower performance. A salient feature of the DRE’s trajectory since 2016 is its marked growth, which contrasts with the trend observed in previous years.

#### 3.2.2 Core independent variable.

Although China has made remarkable achievements in the field of green credit, ranking among the top countries in the world in terms of green credit scale, it is still at a relatively early stage in the overall development process. Currently, there is a lack of direct and precise quantitative indicators to assess the issuance of green credit accurately. Based on many previous research results, the assessment methods of green credit in China mainly cover the following types: First, the Green Credit Guidelines issued by the China Banking Regulatory Commission (CBRC) in 2012 are used as the benchmark for a natural experiment to construct a dummy variable for green credit. Specifically, green credit is characterized by assigning a value of 0 to the period before 2012 and a value of 1 to the period in 2012 and beyond. Second, the ratio between the loan balance of environmental protection-related projects and the total loan balance of financial institutions is calculated as an indicator of the volume of green credit issuance to quantify the scale of green credit from the perspective of the ratio of the loan structure. Thirdly, green credit is regarded as a reverse indicator of the interest expense ratio of the six major energy-intensive industries. With the help of this inverse correlation, an attempt is made to indirectly reflect the development level and scale characteristics of green credit from the relative situation of interest expenses of high-energy-consuming industries. Fourth, the green credit balance and the ratio of loans in “two high and one surplus” (i.e., high-pollution, high-energy-consumption, and overcapacity industries) to all loans are utilized to measure the green credit issuance of major banks to measure the green credit situation in terms of the dimension of the share of loan structure in specific industries. Fifth, the flow of credit resources from polluting to environmentally friendly industries is examined as a measure of green credit, aiming to present the development of green credit from the perspective of transferring credit resources among industries of different natures. Given the careful consideration of various factors such as the completeness and accessibility of data, this study selects the third assessment method mentioned above, i.e., the ratio of the interest expenses of the six major energy-intensive industrial industries in each province to the total interest expenses of the industrial industries in each province is used to measure green credit (GC). The scope of the six high energy-consuming industries has been clearly defined in the 2010 National Economic and Social Development Statistics Report, explicitly covering the chemical raw materials and chemical products manufacturing industry, non-metallic mineral products industry, ferrous metal smelting and calendaring industry, non-ferrous metal smelting and calendaring industry, petroleum processing and coking and nuclear fuel processing industry, and electric power and heat production and supply industry. The corresponding formula is shown below:

GC = (1 − Interest Expenditures of the Six Most Energy−Consuming Industries in Each Province/ Total Interest Expenditures of Industrial Industries× 100%.

#### 3.2.3 Intermediate variables.

**3.2.3.1 Upgrading of industrial structure:** Industrial structure upgrading is a dynamic evolutionary process that promotes the coordinated development of the economy and realizes efficient and reasonable resource allocation through the continuous adjustment of various industries. This paper analyzes the optimization of China’s industrial structure from the dimensions of advanced industrial structure and industrial structure rationalization. For the advanced industrial structure, the calculation formula is as follows:


AISit=∑\nolimitsm=13yi,m,t×lpi,m,t,m=1,2,3lpi,m,t=Yi,m,t/Yi,m,tLi,m,t\nulldelimiterspaceLi,m,t

In the model, $lp_{i,m,t}$ refers to the actual labor productivity level of the industry, and $Y_{i,m,t}$ and $L_{i,m,t}$ represents the value added to the industry, respectively. Value added and the number of employed people respectively. The index can reflect the overall transformation of industrial structure from primary industry to secondary industry to tertiary industry and the qualitative impact of the process of industrial structure upgrading. This paper uses the index to measure the level of advanced industrial structure in different provinces and cities in China.

The rationalization of industrial structure adopts the Thiel index to assess the level of rationalization of industrial structure. The Thiel Index introduces the concept of weighted average, which considers the weight of the output value relative to the GDP. Thus, it is a better measure of the degree of rationalization of industrial structure (IR) than the industrial deviation. Its calculation formula is as follows:


RISit=∑in(YiY)ln(YiLi/YiLiYL\nulldelimiterspaceYL)

When the Tel index is not equal to zero, the industrial structure deviates from the equilibrium state and is irrational. The larger the IR value is, the larger the degree of deviation of the industrial structure is, the worse the efficiency of the industrial structure is, and the more irrational the industrial and employment structure is. The closer the IR value is to zero, the smaller the degree of deviation of the industrial structure is, the better the efficiency of the industrial structure is, the more rational the industrial and employment structure is, and the more mature the industry is.

**3.2.3.2 Green innovation:** Green patents cover invention, design, and utility model patents, with invention patents reflecting high-quality innovation. Green innovation (GINNO) is measured by taking the logarithm of the total number of green patents granted in each province plus one, with higher values indicating more significant innovation in green technologies.

**3.2.3.3 Energy consumption:** Energy consumption (ER) is measured by taking the logarithm of the total amount of coal consumed, with higher values implying greater energy consumption and more pollution of the environment.

#### 3.2.4 Control variables.

Concerning previous literature and to minimize the impact of omitted variables on the research results, this paper considers important control variables that may affect the development of the real economy, and the variables involved are as follows: (1) economic growth (GDPG). Regional economic growth can attract all kinds of necessary human, material, financial, and other factors of production for the development of the real economy, which is crucial for the development of the real economy. The GDP growth rate measures this indicator. Sustained regional economic growth helps to gather various resources, create favorable conditions for the development of the real economy, and promote the improvement of the real economy in terms of scale and quality. (2) Foreign Investment (FDI). Opening- up to the outside world is conducive to China’s enterprises making full use of international resources, introducing advanced technology, driving the improvement of domestic technology level, and then further expanding imports and exports, promoting the development of China’s real economy. The higher the level of opening up to the outside world, the more opportunities there are for exposure to advanced technology, which is more conducive to the progress of the real economy’s technological level, thus promoting the development of the real economy. This indicator is calculated using the ratio of FDI to GDP. The introduction of foreign investment brings not only capital but also advanced management experience and technology, which positively affects the development of the real economy. (3) Education level (EDU). According to Lucas’s human capital spillover theory, human capital has external and internal effects. The improvement of labor productivity of the whole society mainly stems from the external effect of human capital, and it is the existence of this external effect that promotes technological upgrading and social progress, thus promoting economic growth. The improvement of education level can accelerate the progress and accumulation of technology level and management level, which is more conducive to improving real economic efficiency. This indicator is measured by taking the logarithm of the number of general undergraduate and specialized students per 10,000 people in each province. Improving education levels can help cultivate high-quality talents and provide intellectual support for the development of the real economy. (4) Government Investment in Science and Technology (GOV). Increased government investment in science and technology will strongly stimulate innovation in the real industry, which is crucial to developing the real economy. This indicator is calculated by the ratio of local financial expenditure on science and technology to the total local financial expenditure in the general budget. Increased government investment in science and technology can guide enterprises to increase R&D investment and improve the innovation ability and competitiveness of the real economy. (5) Fiscal scale (FISCAL). Fiscal funds are mainly used to support enterprise technological innovation and the provision of public services, which has an apparent pulling effect on the real economy. This indicator is expressed as the ratio of general budget expenditure to GDP. The moderate expansion of the fiscal scale can provide more financial support and policy guarantees for the real economy. (6) Infrastructure (INFRA). Transportation infrastructure mainly includes highways, railroads, airports, ports, bridges, etc., which are important components of the real economy. The indicator is expressed in the number of highway miles per unit of land area. A well-developed infrastructure can reduce logistics costs and improve the operational efficiency of the real economy. (7) Social Consum (CONSUM). Demand determines the willingness of enterprises to develop, and the demand structure can reflect the fundamental role of consumer demand in promoting economic growth. This indicator is calculated from the ratio of total retail sales of consumer goods to GDP. The growth of social demand can stimulate the development of the real economy and encourage enterprises to expand the scale of production and improve the quality of products. The definitions and explanations of the variables are provided in Table 2.

**Table 2:** Definition and explanation of variables.

Types	Variables	Symbols	Definitions
Dependent Variable	Scale of the Real Economy	SRE	GDP per capita in the real economy sector
	Efficiency of the Real Economy	REE	Super-SBM
Independent Variable	Green Credit	GC	The proportion of interest expenses of the six major high-energy-consuming industries in each province to the total interest expenses of the industrial sector
Mediated Variable	High-end Industrial Structure	AIS	The sum of industry gross multiplies the actual labor productivity level of the industry
	Rationalization of Industrial Structure	RIS	Theil index
	Green Innovation	GINNO	Logarithm of the total number of green patent authorizations
	Energy Consumption Structure	ER	Logarithm of total coal consumption
Control Variable	economic Growth	GDPG	GDP Growth Rate
	Foreign Investment	FDI	Foreign Direct Investment (FDI) as a percentage of GDP
	Level of Education	EDU	Logarithm of the Number of Regular Undergraduate Students per 10,000 People by Province
	Government Investment in Science and Technology	GOV	Local government science and technology expenditure as a percentage of total budget
	Fiscal Scale	FISCAL	General budget expenditure as a percentage of GDP
	Infrastructure	INFRA	Road Milestone per Unit Area of Land
	Social Consum	CONSUM	Retail sales as a percentage of GDP

#### 3.2.5 Data and descriptive statistics.

The period from 2007 to 2022 was selected as the sample interval, incorporating 30 provinces, municipalities, and autonomous regions in China, and a total of 480 sample observations were obtained. Data on the total output value of the real economy in each province and city were obtained from the China Statistical Yearbook of the past years. Data on labor input and fixed asset investment in the real economy are obtained from the China Statistical Yearbook, the China Labor Statistical Yearbook, the China Population and Employment Statistical Yearbook, the China Fixed Asset Investment Statistical Yearbook, and the Wind Information Database, respectively. Data on green credit is taken from the China Industrial Statistics Yearbook. Data on other control variables: Other control variables are sourced from the National Bureau of Statistics (NBS) official website, the Wind Information Database, and the statistical yearbooks of provinces (autonomous regions and municipalities directly under the central government). For individual missing data, linear interpolation is used as a supplement. To alleviate the problems of extreme values and heteroskedasticity, the absolute data are logarithm zed in this study to eliminate the magnitude error; meanwhile, the price data are treated as constant with 2003 as the base period. The descriptive statistics are shown in Table 3. The mean of GC is 0.516, and the standard deviation is 0.145, indicating that the average GC in our sample is high and there are significant differences. Control variables are consistent with previous studies.

**Table 3:** Description of variables.

Type	Variables	Obs	Mean	Std. Dev.	Min.	Max.
Dependent variable	SRE	480	3.131	1.766	0.581	10.281
	REE	480	0.516	0.184	0.236	1.034
Core independent variable	GC	480	0.449	0.145	0.094	0.771
Control variables	GDPG	480	8.941	3.851	5.000	19.200
	FDI	480	0.022	0.021	0.000	0.121
	EDU	480	5.818	0.230	5.139	6.312
	GOV	480	0.021	0.015	0.004	0.072
	FISCAL	480	0.246	0.108	0.097	0.758
	INFRA	480	11.508	0.852	8.994	12.982
	CONSUM	480	0.384	0.065	0.183	0.610

## 4. Empirical results and analysis

### 4.1 Benchmark regression results

It is acknowledged that fixed effects may substantially influence the coefficients and significance; therefore, this paper employs a regional time fixed effects model for estimation. Utilizing models (1) and (2), Table 5 presents the estimation results of the benchmark model. The results reveal a significant positive correlation between green credit and the size of the real economy, indicating that the development of green credit helps to promote the expansion of the size of the regional real economy. However, a significant negative correlation between green credit and real economic efficiency is also revealed, implying that it fails to improve the efficiency of the regional real economy effectively and may even have a specific inhibitory effect on it.

**Table 5:** Robustness checks results.

Variables	Tobit stratege		Winsorizing at level 1 % and 99 %		Delete after 2017		Exclusion of COVID-19 impact		GLS	
	(1)	(2)	(3)	(4)	(5)	(6)	(7)	(8)	(9)	(10)
	SRE	REE	SRE	REE	SRE	REE	SRE	REE	SRE	REE
GC	0.666^***^	−0.155^***^	0.658^***^	−0.156^***^	0.481^*^	−0.136^***^	0.602^**^	−0.140^***^	.348^***^	−0.097^***^
	(0.236)	(0.033)	(0.238)	(0.035)	(0.255)	(0.047)	(0.239)	(0.038)	(0.123)	(0.023)
Controls	Yes	Yes	Yes	Yes	Yes	Yes	Yes	Yes	Yes	Yes
Province	Yes	Yes	Yes	Yes	Yes	Yes	Yes	Yes	Yes	Yes
Year	Yes	Yes	Yes	Yes	Yes	Yes	Yes	Yes	Yes	Yes
Constant	16.677^***^	2.198^***^	16.137^***^	1.837^***^	14.317^***^	1.478^***^	15.895^***^	1.786^***^	11.301^***^	1.293^***^
	(1.410)	(0.208)	(1.507)	(0.221)	(1.427)	(0.260)	(1.517)	(0.239)	(1.214)	(0.182)
Observations	480	480	480	480	330	330	390	390	480	480
Adjusted R-squared	–	–	0.971	0.943	0.978	0.946	0.972	0.944	–	–
F	–	–			11.95	8.12	15.08	11.06	–	–

The initial two columns of Table 4 illustrate the impact of green credit on the size and efficiency of the real economy in the absence of control variables. The findings indicate that the estimated coefficient of green credit on the size of the real economy is positive at the 1% significance level, with a value of 0.930. In comparison, the effect on the efficiency of the real economy is negative at the 1% significance level, with a coefficient of −0.129. Columns (3) and (4) illustrate the impact of green credit on the size and efficiency of the real economy, with the inclusion of control variables. The estimation results continue to demonstrate that the impact of green credit on the size of the real economy is positive at the 1% significant level, with a coefficient of 0.666, and the impact on the efficiency of the real economy is negative at the 1% significant level, with a coefficient of −0.155. This further corroborates the hypothesis that green credit positively influences the growth of the real economy, albeit with certain limitations in terms of enhancing its efficiency.

**Table 4:** Benchmark regression results.

Variables	(1)	(2)	(3)	(4)
	SRE	REE	SRE	REE
GC	0.930^***^	−0.129^***^	0.666^***^	−0.155^***^
	(0.286)	(0.039)	(0.251)	(0.035)
GDPG			−0.006	0.006^***^
			(0.011)	(0.002)
FDI			−5.585^***^	−1.087^***^
			(1.307)	(0.1813)
lnEDU			−2.189^***^	−0.218^***^
			(0.229)	(0.032)
GOV			1.301	1.208^***^
			(3.241)	(0.450)
FISCAL			−1.466^**^	−0.189^**^
			(0.594)	(0.082)
INFRA			0.018	−0.010
			(0.099)	(0.014)
SD			0.193	0.102^*^
			(0.3845)	(0.053)
Province	Yes	Yes	Yes	Yes
Year	Yes	Yes	Yes	Yes
Constant	2.713^***^	0.5739^***^	15.770^***^	1.921^***^
	(0.130)	(0.0175)	(1.619)	(0.225)
Observations	480	480	480	480
Adjusted R-squared	0.956	0.926	0.967	0.942
F	10.59	11.19	19.88	16.31

Note: t-values are in parentheses. *, ** and *** Table show the statistical significance levels of 10%, 5% and 1%, respectively. The same note applies below.

The empirical finding that green credit significantly reduces the efficiency of the real economy reveals that, in the current institutional context of China, there is a lack of adequate systemic safeguards and market mechanisms to ensure that green credit effectively enhances the efficiency of the real economy. We will conduct an in-depth exploration of the underlying mechanisms in the mechanism analysis section.

Furthermore, the regression results of the control variables in columns (2) and (4) of Table 4 demonstrate that economic growth and government investment in science and technology have a significant positive effect on the enhancement of the efficiency of the real economy of the region, with regression coefficients of 0.006 and 1.208, respectively. This indicates that the rapid growth of the regional economy, as well as the increase in government investment in science and technology, can effectively contribute to the enhancement of the efficiency of the real economy. However, the regression coefficients of education level, government intervention, and foreign direct investment are significantly negative, indicating that these factors are ineffective in promoting the improvement of the scale and efficiency of the real economy. The underlying reasons for this phenomenon are multifaceted and include: Firstly, the current employment situation in China is challenging, necessitating an urgent adjustment to the employment structure, which in turn has a detrimental effect on the efficiency of the real economy. Secondly, despite the rapid increase in higher education in China, many highly educated individuals have remained unemployed for extended periods due to the constraints of the employment environment, resulting in the under-utilization of their educational advantages. Finally, government intervention may impede the market-oriented development of the economy, thereby hindering the growth of the real economy.

### 4.2 Robustness checks

#### 4.2.1 Alternative estimation model.

In the observations of the present study, the dependent variable assumes values over 0. In such cases, employing the ordinary least squares (OLS) method may result in biased parameter estimation. To reduce the left-censored bias in the sample, the Tobit method was used for estimation in this study. As demonstrated in columns (1) and (2) of Table 5, green credit has been shown to exert a significant positive effect on the size of the real economy while concurrently demonstrating a significant adverse effect on the efficiency of the real economy. This finding further substantiates the robustness and credibility of the study’s findings.

#### 4.2.2 Winsorization.

To circumvent the potential interference of outliers with the results, the sample data in this study underwent 1% two-sided shrinkage. The results presented in columns (3) and (4) of Table 5 demonstrate that the sign and significance of the regression coefficients of green credit on the development of the real economy remain constant. The regression coefficients following the two-sided shrinkage treatment were determined to be 0.6579 and −0.1555, respectively, exhibiting minimal deviation from the overall regression coefficients. This finding underscores the reliability and robustness of the regression results.

#### 4.2.3 Changing the sample interval.

In 2017, the scope of national low-carbon pilot cities was expanded further, and the first batch of state-level green financial reform and innovation pilot zones was established in the same year. To exclude the possible impact bias of these policies, this study excludes the data samples after 2017 and re-runs the regression analysis. The regression results are displayed in columns (5) and (6) of Table 5. The estimation results presented herein serve further to corroborate the robustness of the benchmark regression results.

#### 4.2.4 Exclusion of COVID-19 impact.

In early 2020, production activities were extensively restricted to prevent and control the public safety impact of the outbreak of the novel COVID-19, leading to a sharp decline in consumer demand and economic growth. To cope with the impact of this unprecedented public health event, the study excluded the sample for 2020 and beyond and re-estimated the regression model. The results in columns (7) and (8) of Table 5 demonstrate that the estimates of green credit on the size and efficiency of the real economy remain significant even when the impact of the outbreak is considered, thus validating the results of the benchmark regression.

#### 4.2.5 Generalized least squares.

We perform a test of homoskedasticity and find that there is a problem with heteroskedasticity. Therefore, we use the GLS method to regress, and the regression results are shown in columns 9 and 10 of Table 5. It can be seen that after dealing with issues such as heteroskedasticity, our results are still robust.

#### 4.2.6 Endogeneity test.

Given the variations in green credit policies across different regions, the size and efficiency of the real economy may also be impacted. In such a scenario, green credit ceases to be a purely exogenous random variable, and it may give rise to endogeneity concerns, which may hinder the precision of regression coefficient estimation and result in biased analysis outcomes. This paper proposes a two-stage least squares (2SLS) regression analysis with carefully selected instrumental variables to address this challenge.

We have constructed two instrumental variables for green credit. The first is the lagged first-order term of green credit, and the second is the interaction between the shortest distance to the coastal port t and the total amount of loans of financial institutions across the country in the previous year. For the first instrumental variable, considering the policy continuity of each province, the lagged first-order term of green credit is highly correlated with the current scale of green credit, thus meeting the correlation condition. Green credit is generally utilized in the current year and does not directly affect future economic development, fulfilling the exclusivity condition. For the second instrumental variable, a shorter distance to the coastal port indicates a higher level of economic development in that region, a more complete organizational structure, and an improved institutional environment, which is more conducive to the development of green finance, meeting the correlation condition. Furthermore, the distance to the coastal port is unlikely to have a significant direct impact on the development of regional new quality productivity, thus meeting the exogeneity condition. The regression results for the two instrumental variables are presented in columns 1–2 and 3–4 of Table 6, respectively.

**Table 6:** Endogeneity test.

Variables	IV1		IV2	
	(1)	(2)	(3)	(4)
	SRE	REE	SRE	REE
GC	1.582^***^	−0.283^***^	0.951^***^	−0.294^***^
	(0.462)	(0.068)	(0.293)	(0.083)
Constant	16.677^***^	2.198^***^	18.203^***^	1.859^***^
	(1.410)	(0.208)	(1.614)	(0.342)
Controls	Yes	Yes	Yes	Yes
Province	Yes	Yes	Yes	Yes
Year	Yes	Yes	Yes	Yes
Kleibergen-Paap rk Wald F statistic	55.546		81.482	
Kleibergen-Paap rk LM statistic	85.962^***^		79.364^***^	
Cragg-Donald Wald F	193.56		144.392	
Observations	450	450	450	450
R-squared	0.909	0.581	0.924	0.591

The analysis demonstrates a significant positive correlation between green credit and the size of the real economy. In contrast, a significant negative correlation is observed with the efficiency of the real economy at the 1% significance level. The hypothesis of unidentifiable and weak instrumental variables is rejected, indicating the validity of the selected instrumental variables and the robustness of the regression results. It is acknowledged that endogeneity issues, such as omitted variables, can potentially induce estimation bias.

Therefore, column (3) constructs the interaction term between green credit and the number of outlets per 10,000 people in the sample area in the previous year as a national ratio as an instrumental variable. The findings indicate that at the 10% significance level, a substantial positive correlation persists between green credit and the magnitude of the real economy, concurrently with a notable negative correlation to the efficiency of the real economy. Furthermore, the null hypotheses of unidentifiable and weak instrumental variables were rejected, confirming the validity of the selected instrumental variables and the reliability of the regression results. Furthermore, the endogeneity test and Kleibergen-Paaprk LM test rejected the original hypothesis at the 1% level of significance, and the Kleibergen-Paaprk Wald F statistic was found to be greater than the critical value of the Stock-Yogo test at the 10% level. This finding reinforces the validity of the instrumental variables employed and dispels any concerns regarding their potential for identification and weakness. The two-stage least squares regression findings further substantiate these outcomes, accentuating the positive impact of green credit on the size of the real economy while concurrently highlighting its negative impact on the efficiency of the real economy. This finding suggests that green credit can promote economic growth when potential endogeneity concerns are considered, though it may impede efficiency.

## 5. Further analyses

### 5.1 Mechanism analyses

#### 5.1.1 Industrial structure upgrading.

This study provides an in-depth analysis of the mediating effect of green credit in promoting industrial structure upgrading. The results demonstrate that green credit positively influences the growth of the real economy by promoting the industrial structure in a more advanced direction, as illustrated in columns (1)-(3) of Table 7. However, the analysis in columns (4)-(6) of Table 7 reveals the impact of green credit on rationalizing industrial structure. Although it shows a significant positive effect since the coefficient of industrial structure rationalization is a negative indicator, this indicates that green credit hurts the rationalization of industrial structure. In addition, the significant positive effect of industrial structure rationalization on real economic development has not been confirmed.

**Table 7:** Mechanism analysis: industrial structure upgrading mechanism.

Variables	(1)	(2)	(3)	(4)	(5)	(6)
	AIS	SRE	REE	RIS	SRE	REE
GC	0.184^*^		−0.169^***^	3.611^**^		
	(1.85)		(−4.90)	(2.28)		
AIS		1.605^***^	0.046^***^			
		(17.29)	(2.76)			
RIS					0.009	0.001
					(1.13)	(0.94)
Controls	Yes	Yes	Yes	Yes	Yes	Yes
Province	Yes	Yes	Yes	Yes	Yes	Yes
Year	Yes	Yes	Yes	Yes	Yes	Yes
Constant	6.143^***^	6.558^***^	1.538	37.807^***^	16.094	1.783^***^
	(10.04)	(5.02)	(6.56)	(3.88)	(10.34)	(8.25)
Observations	480	480	480	480	480	480
Adjusted R-squared	0.943	0.981	0.943	0.849	0.967	0.942
F	22.31	66.64	17.17	5.62	19.85	16.17

One potential explanation for this phenomenon is that green credit should be analyzed from the perspective of its capital formation mechanism. This would restrict the ability of polluting enterprises to obtain credit by directing the flow of funds to green and clean industries while raising the financing costs of those enterprises. This differentiated credit policy may cause some highly polluting and energy-intensive enterprises to encounter financial difficulties, which would inhibit the expansion of these industries and thus adversely affect the rationalization of industrial structure. Secondly, the contradictions and problems arising from the urban-rural dual structure have become increasingly prominent, and the rationalization of industrial structure has been affected by the distortion of factor allocation. Enterprises may not be able to make timely adjustments in response to market changes, making it difficult to improve the efficiency of resource allocation, which in turn affects the overall scale and efficiency of the economy.

#### 5.1.2 Green innovation.

The first column of Table 8 examines the relationship between green credit and green innovation. The results show that there is a significant negative correlation between the two. This finding suggests that current green credit funds may not be efficiently allocated to green innovation projects with positive externalities. Instead, there is a possibility that these funds may have a crowding-out or inhibiting effect, resulting in the “crowding-out effect” caused by green credit exceeding its “positive spillover.” Furthermore, the investigation suggests that green finance may exhibit spatial spillovers, potentially diminishing the direct impact of green credit on local green innovation. The empirical result that green credit may inhibit corporate green innovation indicates the absence of a robust policy and regulatory framework, as well as effective market mechanisms, to support the efficient operation of green credit in China. Consequently, the allocation of green credit resources both across firms and within individual firms—among various projects—remains inefficient.

**Table 8:** Mechanism analysis: green innovation.

Variables	(1)	(2)	(3)
	GINNO	SRE	REE
GC	−0.711^***^		
	(4.04)		
GINNO		−0.273^***^	0.026^***^
		(−4.07)	(2.80)
Controls	Yes	Yes	Yes
Province	Yes	Yes	Yes
Year	Yes	Yes	Yes
Constant	3.402^***^	17.348 ^***^	1.731^***^
	(3.14)	(11.41)	(8.12)
Observations	480	480	480
Adjusted R-squared	0.981	0.969	0.943
F	11.06	22.25	17.20

Columns (2)-(3) of Table 8 examine the impact of green innovation on the size and efficiency of the real economy, respectively. The results indicate that green innovation positively influences the efficiency of the real economy but simultaneously exhibits a mitigating effect on its size. This phenomenon may be attributed to the fact that green innovation typically necessitates substantial capital and resource investments, and green credit may accord priority to allocating these resources to environmentally sustainable initiatives during the resource allocation process. Consequently, the funds available to traditional industries are correspondingly diminished, thereby impeding the growth of specific energy-intensive industries and exerting an indirect influence on the size of the real economy. Nevertheless, this transition is conducive to enhancing resource utilization efficiency and productivity in the long run.

#### 5.1.3 Energy consumption.

The first column of Table 9 examines the relationship between green credit and energy consumption. It demonstrates that the effect of green credit on energy consumption is significantly negative at the 1% level. This suggests that an increase in the scale of green credit can significantly reduce energy consumption. This finding suggests that green credit encourages enterprises to adopt more energy-efficient and environmentally friendly production methods by offering favorable loan terms, providing support for clean energy projects, and adopting energy-efficient technologies. Consequently, this results in a reduction in dependence on traditional energy sources and a decrease in the overall intensity of energy consumption.

**Table 9:** Mechanism analysis: energy consumption.

Variables	(1)	(2)	(3)
	ER	SRE	REE
GC	−0.718^***^		
	(−5.534)		
ER		−0.541^***^	−0.006
		(−6.239)	(−0.450)
Controls	Yes	Yes	Yes
Province	Yes	Yes	Yes
Year	Yes	Yes	Yes
Constant	5.527^***^	19.788^***^	1.652^***^
	(6.935)	(13.158)	(7.464)
Observations	480	480	480
Adjusted R-squared	0.960	0.970	0.939
F	10.06	27.54	14.70

Columns (2) and (3) of Table 9 examine the impact of energy consumption intensity on the size and efficiency of the real economy, respectively. The findings indicate that energy consumption significantly negatively influences the size of the real economy at the 1% level. In contrast, its impact on the efficiency of the real economy is negative but not significant. One potential explanation for this phenomenon could be that increased energy consumption ultimately leads to elevated production costs and hinders the growth of firms’ production scale. This, in turn, may diminish firms’ competitiveness and consequently exert an influence on the size growth of the real economy. However, the non-significant impact on the efficiency of the real economy may be attributable to energy-saving measures or technological improvements adopted by enterprises, such as enhancing energy efficiency, utilizing energy-efficient equipment, or optimizing production processes, which to some extent offset the negative impact of higher energy costs.

### 5.2 Heterogeneity analyses

#### 5.2.1 Regional heterogeneity.

A multi-factor analysis, incorporating geopolitical and economic development, has led to the division of China’s 30 provincial administrative units into three regions: namely, eastern, central, and western. This division has been undertaken based on the regional classification criteria established by the National Bureau of Statistics of China. The regression analysis results are presented in Table 10. The analysis reveals that green credit in the western region significantly promotes the expansion of the real economy scale and the improvement of real economy efficiency; the development of green credit in the eastern region only has a significant positive impact on the expansion of the real economy scale, but has an inhibitory effect on real economy efficiency; and the green credit in the central region does not show a significant promotional effect in terms of both the real economy scale and efficiency. The following hypotheses may provide a basis for explaining these phenomena: firstly, the eastern region is significantly ahead of the central and western regions in terms of industrial structure, technological level, and the degree of development of the financial system, which makes the scale of green credit in this region more prominent and thus has a significant role in promoting the scale of the real economy. However, it is important to note that the rapid economic development in the eastern region has resulted in a more sophisticated financing system, which has led to a relatively large scale of green credit. This has led to the marginal effect of the economy being saturated and the possibility of a crowding-out effect, which could ultimately impede the efficiency of the real economy. By contrast, the central region exhibits a comparatively lower level of financial development, with economic growth predominantly reliant on heavy industry. Concurrently, relocating industries from developed coastal regions to the central region has precipitated a surge in environmental pollution. The region’s industrial layout and investment orientation appear to deviate from the principles of sustainable development, resulting in the development of green credit being comparatively deficient. The implementation of green credit, therefore, may potentially impede the growth of the real economy. In response to these challenges, the central government has implemented a Western development strategy, allocating resources to the Western region and attracting various factors, thereby creating favorable conditions for its economic development. The implementation of more stringent environmental regulations in the eastern region has prompted the relocation of certain polluting enterprises to the western region, where environmental regulations are less stringent. Green credit, as a nationwide environmental regulation policy, ensures that these enterprises cannot evade compliance any longer and must instead self-adjust, leading to the reorganization of industrial structure and the enhancement of green total factor productivity. This, in turn, promotes the development of the Western region’s economy more broadly. Furthermore, as the western region continues to experience increased urbanization and economic growth, there is an attendant rise in the importance of ecological environmental protection and construction, thereby promoting the coordinated development of the economy and the environment. The development of green credit aligns with the trend of green transformation of the western region’s economy and significantly promotes the development of the real economy.

**Table 10:** Heterogeneity of region.

	East		Center		West	
Variables	SRE	REE	SRE	REE	SRE	REE
GC	1.693^***^	−0.262^***^	−0.100	−0.035	0.719^*^	0.309^***^
	(0.41)	(0.06)	(0.23)	(0.046)	(0.41)	(0.09)
Controls	Yes	Yes	Yes	Yes	Yes	Yes
Province	Yes	Yes	Yes	Yes	Yes	Yes
Year	Yes	Yes	Yes	Yes	Yes	Yes
Constant	5.52	2.81^***^	9.75^***^	−0.90^**^	12.06^***^	1.92^***^
	(3.42)	(0.49)	(2.01)	(0.40)	(1.77)	(0.37)
Observations	176	176	128	128	176	176
Adjusted R-squared	0.97	0.94	0.98	0.96	0.97	0.91

#### 5.2.2 Heterogeneity in the degree of development of the banking system.

The findings of the regression analysis presented in Table 11 indicate that, in fostering the advancement of the real economy, green credit shows a more substantial impact in regions with more developed banking systems. The specific data demonstrate that the correlation coefficients reach 0.948 and −0.277, respectively, and are statistically significant at the 1% level. This finding can be attributed to the fact that regions with well-developed banking systems tend to have more advanced banking markets, which can provide better regional environmental infrastructure and substantial financial support. Consequently, in these regions, green credit can optimize fund allocation and encourage enterprises to increase R&D investment and expand production scale, thereby promoting the growth of the real economy. At the same time, the inhibitory effect of green credit on the efficiency of the real economy has been significantly mitigated.

**Table 11:** Heterogeneity of degree of development of the banking system.

Variables	Developed	Development Central	Developed	Development Central
	SRE	SRE	REE	REE
GC	1.143^***^	−0.298	−0.063	−0.256^***^
	(0.341)	(0.250)	(0.048)	(0.046)
Controls	Yes	Yes	Yes	Yes
Province	Yes	Yes	Yes	Yes
Year	Yes	Yes	Yes	Yes
Adjusted R-squared	0.979	0.981	0.934	0.971
Observations	240	238	238	240

#### 5.2.3 Heterogeneity in environmental regulation.

Research has demonstrated that government environmental regulations can incentivize firms to optimize their production processes and reduce pollution emissions to comply with environmental standards. Moreover, environmental regulation is pivotal in encouraging firms to engage in green technological innovation and promoting green innovation development. Furthermore, environmental regulation may motivate firms to increase green investment through social and moral pressure from the public. In this study, we selected the frequency of environment-related terms in local government work reports as a proxy variable for environmental regulation (GER). The study sample was then categorized into regions with higher and lower environmental regulation based on the annual median.

The regression analysis findings presented in Table 12 indicate that the estimated coefficient of green credit on the size of the real economy is positive. However, it is statistically insignificant in regions with stricter environmental regulations. Conversely, in regions characterized by less stringent environmental regulations, green credit is positively associated with the size of the real economy at the 1% level. Moreover, green credit has been observed to exhibit a significant negative association with real economic efficiency (REE). This finding suggests that environmental regulation moderates the relationship between green credit and the real economy. Specifically, in regions characterized by strict environmental regulations, green credit exerts a negligible effect on the size of the real economy, presumably because firms have already incurred substantial environmental compliance costs in such regions, thereby rendering the marginal effect of green credit relatively insignificant and failing to elicit a substantial change in their production behavior. Conversely, the positive impact of green credit on the size of the real economy is more significant in regions with lower levels of environmental regulation, presumably because green credit provides more financial support to firms, encouraging them to expand their production scale and adopt more environmentally friendly technologies. However, due to the relatively lax environmental regulations in these regions, firms may be more inclined to pursue short-term economic benefits, which may consequently lead to a reduction in the efficiency of the real economy.

**Table 12:** Heterogeneity of environmental regulation.

Variables	Developed	Developing	Developed	Developing
	SRE	SRE	REE	REE
GC	0.273	1.144^***^	−0.129^**^	−0.144^***^
	(0.741)	(3.008)	(−2.433)	(−2.610)
Controls	Yes	Yes	Yes	Yes
Province	Yes	Yes	Yes	Yes
Year	Yes	Yes	Yes	Yes
Constant	20.417^***^	11.490 ^***^	2.310^***^	1.273 ^***^
	(8.99)	(6.34)	(7.24)	(5.04)
Adjusted R-squared	0.966	0.974	0.941	0.945
Observations	248	229	248	229

## 6. Conclusions and policy recommendations

This paper adopts the panel data of 30 provinces in China from 2007 to 2020 and further empirically examines the dual impact of green credit on the scale and efficiency of the real economy by using the two-way fixed panel method on the theoretical analysis of the mechanism of green credit affecting the development of the real economy. The research results show that: First, green credit has a significant expansion effect on the scale of the real economy, but the enhancement effect on the efficiency of the real economy presents a complex situation, the overall enhancement effect is limited, and in some cases, there is even an inhibitory effect. Second, green credit on the development of the real economy promotes the effect in the Western region; the banking system is more developed, and environmental regulations are more relaxed in the region more obvious. Third, green credit mainly affects the real economy by promoting optimization, upgrading industrial structure, and reducing energy consumption. However, the path to green innovation has not yet played the expected effect in promoting the development of the real economy. Based on these findings, we propose the following policy recommendations:

(1) Specialized support for industrial energy efficiency improvement. The government and financial institutions should work together to set up a “special credit for industrial energy efficiency improvement” for industries in the real economy with high energy consumption but with the potential for transformation, such as the traditional manufacturing industry. The credit funds are specifically used to support enterprises in introducing advanced energy-saving and emission-reduction technologies and equipment and carrying out energy management system construction projects. At the same time, a professional energy efficiency consulting team provides enterprises with a full range of guidance from technology selection to project implementation to ensure that the green credit funds are effectively transformed into energy efficiency to improve the momentum and thus improve the efficiency of the real economy.
(2) Incentivize green supply chain finance. Incentivize financial institutions to develop green supply chain financial services and provide more favorable green credit interest rates and more significant credit limits for core enterprises that build green supply chains and promote upstream and downstream enterprises to jointly carry out green production transformation. Through the leading role of core enterprises, small and medium-sized enterprises in the industrial chain can be promoted to jointly improve green production, optimize resource allocation, and reduce waste and inefficiency in the production process to enhance the real economy’s operational efficiency comprehensively.
(3) Create green industry clusters in the western region. In addition to establishing special credit funds, local governments should also plan green industrial parks and provide preferential land-use policies and taxation for green enterprises entering the parks. Financial institutions should be built around the park to provide a “one-stop” green financial service center, integration of green credit, venture capital, industrial funds, and other financial tools to attract relevant upstream and downstream enterprises to form a complete green industry cluster. This can maximize the role of green credit in promoting the development of the real economy in the Western region and consolidate the foundation of industrial development.
(4) Lead the innovation in developed areas of the banking system. Banks should improve their monitoring of post-green-credit projects, particularly those related to green innovation that require a longer time horizon to yield returns, and should consider providing green credit based on the long-term performance of such green innovations. Support the deep cooperation between banks and financial technology enterprises to develop a green credit risk assessment and accurate marketing system based on big data and blockchain technology. On the one hand, big data technology is utilized to accurately identify enterprises with green potential and intervene in advance to provide credit support; on the other hand, blockchain technology is used to ensure the traceability of the flow of green credit funds and improve the accuracy of capital allocation. At the same time, a green financial innovation laboratory is established to gather the strength of all parties in industry, academia, and research to research and develop new types of green financial products, such as green asset-backed securities, etc., to continue to promote the innovation of green credit and inject new vitality into the real economy.
(5) Guide the transformation of regions with lax environmental regulations. Local governments and industry associations should regularly release reports on the development trend of green industries and guide enterprises to lay out high-value-added and low-pollution emerging green industries in advance. Based on these reports, financial institutions should formulate personalized green credit transformation programs for enterprises and set phased credit investment targets and assessment indicators. With the gradual transition of enterprises to green industries, financial institutions should increase credit support to ensure a smooth transition of enterprises in the dynamic adjustment of environmental regulations and continue to promote the development of the real economy.
(6) Build a risk-sharing mechanism for green innovation. The government, financial institutions, and enterprises can co-finance establishing a “green innovation risk compensation fund” according to a certain percentage to provide a certain percentage of loss compensation when the enterprise’s green technology innovation project fails to reduce the risk of enterprise innovation concerns. At the same time, under the protection of the risk compensation fund, financial institutions can appropriately relax the credit conditions for green innovation projects, thereby enhancing the scope of green credit support for innovation projects.
(7) Optimize green innovation intellectual property pledge financing. Improve the intellectual property assessment system and introduce professional green technology intellectual property assessment organizations to improve the accuracy and credibility of the assessment. Financial institutions can innovate intellectual property pledge financing products accordingly and provide higher pledge rates and more flexible repayment options for enterprises with core intellectual property rights for green innovation. This will broaden the funding channels for enterprises’ green technology research and development and accelerate the output and application of green innovation results.

However, due to data availability, this paper faces certain limitations. Although this study provides an in-depth analysis of the relationship between green credit and the real economy, there are some limitations. First, the sample is limited to provincial data in China, which may not fully capture the impact of green credit on a global scale. Second, there may be some bias in the data sources, affecting the results’ universality. Future research could consider cross-country data to enhance the universality of the findings. In addition, future research could explore the heterogeneity of the impact of green credit on the real economy in different industries and the spatial impact of green credit on the real economy.
